# The Effects of Restriction Pressures on the Acute Responses to Blood Flow Restriction Exercise

**DOI:** 10.3389/fphys.2019.01018

**Published:** 2019-08-13

**Authors:** Michael J. Ilett, Timo Rantalainen, Michelle A. Keske, Anthony K. May, Stuart A. Warmington

**Affiliations:** ^1^School of Exercise and Nutrition Sciences, Institute for Physical Activity and Nutrition, Deakin University, Geelong, VIC, Australia; ^2^Gerontology Research Center, Faculty of Sport and Health Sciences, University of Jyväskylä, Jyväskylä, Finland

**Keywords:** Kaatsu, blood flow restriction, muscle fatigue, EMG, limb occlusion pressure, restriction pressure

## Abstract

**Purpose:**

No current guidelines or recommendations exist informing the selection of restriction pressure during blood flow restriction exercise (BFRE). Moreover, the effects of specific relative restriction pressures on the acute muscle, metabolic and cardiopulmonary responses to BFRE are unclear. The purpose of this study was to characterize these acute responses at different levels of restriction pressure.

**Methods:**

Participants (*n* = 10) completed rhythmic isometric knee extension exercise across five experimental trials in a balanced randomized order. Three were BFRE trials {B-40 [restriction pressure set to 40% LOP (total limb occlusion pressure)]; B-60 (60% LOP); and B-80 (80% LOP)} with a workload equivalent to 20% maximal voluntary force (MVC), one was non-BFRE at 20% MVC (LL) and one was non-BFRE at 80% MVC (HL). Measurements recorded were torque, muscle activity via electromyography (EMG), tissue oxygenation via near infrared spectroscopy, whole body oxygen consumption, blood lactate and heart rate.

**Results:**

For the LL and B-40 trials, most measures remained constant. However, for the B-60 and B-80 trials, significant fatigue was demonstrated by a reduction in MVC torque across the trial (*p* < 0.05). Blood lactate increased from baseline in HL, B-60, and B-80 (*p* < 0.05). Submaximal EMG was greater in B-60 and B-80 than LL, but lower compared with HL (*p* < 0.05). Tissue oxygenation decreased in HL, B-40, B-60, and B-80 (*p* < 0.05), which was lower in the B-80 trial compared to all other trials (*p* < 0.01). Whole body oxygen consumption was not different between the BFRE trials (*p* > 0.05).

**Conclusion:**

We demonstrate graded/progressive acute responses with increasing applied pressure during BFRE, from which we speculate that an effective minimum “threshold” around 60% LOP may be necessary for BFRE to be effective with training. While these data provide some insight on the possible mechanisms by which BFRE develops skeletal muscle size and strength when undertaken chronically across a training program, the outcomes of chronic training programs using different levels of applied restriction pressures remain to be tested. Overall, the present study recommends 60–80% LOP as a suitable “minimum” BFRE pressure.

## Introduction

To increase skeletal muscle size and strength it is recommended to lift loads that exceed 65–70% one repetition maximum (1-RM) (American College of Sports Medicine, 2009). However, exercise training using relatively light intensities [20–30% of maximal voluntary contraction (MVC)] in combination with an externally applied blood flow restriction (BFR) can elicit similar gains in skeletal muscle size and strength compared with traditional heavy-load resistance exercise (HLRE) (Shinohara et al., 1997; Karabulut et al., 2010; Yasuda et al., 2011). This light-intensity blood flow restriction exercise (BFRE) has significant practical application for a range of population groups that may be contraindicated to perform HLRE, such as older adults (Abe et al., 2010; Karabulut et al., 2010), athletes and/or patients recovering from musculoskeletal conditions such as anterior cruciate ligament injuries (Takarada et al., 2000b; Ohta et al., 2003). However, the restriction pressures applied during BFRE have historically been arbitrarily selected, and most often based on pressures used previously (Fahs et al., 2011; Takada et al., 2011; Hunt et al., 2013; Neto et al., 2016). This is independent of whether an absolute pressure is selected (Fahs et al., 2011; Hunt et al., 2013; Karabulut et al., 2013), or whether the pressure selected is individualized to structural and physiological characteristics of participants (Cook et al., 2007; Laurentino et al., 2012; Loenneke et al., 2013). Consequently, it so happens that few studies have attempted to examine the influence of different magnitudes of restriction pressure on acute responses to BFRE (Yasuda et al., 2008; Fatela et al., 2016), that may in turn influence the magnitude of any gains in skeletal muscle size and strength with chronic BFRE training.

A demonstrated acute response to BFRE is the greater rate of acute fatigue development than equivalent intensity light-load resistance exercise (LLRE) (Wernbom et al., 2008; Farup et al., 2015; Yasuda et al., 2015). However, until recently (Fatela et al., 2016), this has only been examined using single arbitrary pressures (Wernbom et al., 2008; Manimmanakorn et al., 2013; Farup et al., 2015). This heightened fatigue aligns with demonstrations of elevated skeletal muscle electromyography (EMG) activity across a BFRE bout (Wernbom et al., 2008; Yasuda et al., 2008, 2013; Fatela et al., 2016), leading many to speculate that this fatigue is compensated by increased muscle activation (Takarada et al., 2000c; Yasuda et al., 2008; Fatela et al., 2016). This elevated EMG activity is suggested to arise from increased Type II muscle fiber recruitment with BFRE (Takarada et al., 2000c; Fatela et al., 2016), and as such it has been speculated that Type II fibers are the primary source of the overall increased strength and muscle growth following chronic BFRE training (Loenneke et al., 2014; Pearson and Hussain, 2015). However, this has recently been challenged following 6.5 weeks BFRE training in powerlifters that showed greater proliferation of myonuclei and expansion of fiber area in Type I compared with Type II muscle fibers (Bjørnsen et al., 2018), although again this study used only a single arbitrary pressure.

The proposed greater type II fiber recruitment in response to BFRE has previously been reinforced by a greater accumulation of blood lactate (BLa) (Takarada et al., 2000c; Takano et al., 2005; Kim et al., 2014; Staunton et al., 2015), which inherently arises when Type II fibers are active (Pearson and Hussain, 2015). This suggests greater muscle acidity during BFRE compared with LLRE that likely stimulates growth hormone (GH) release (Takarada et al., 2000a; Takano et al., 2005; Fujita et al., 2007), which may induce subsequent muscle hypertrophy contributing to the increase in strength with chronic training (Takarada et al., 2000c; Abe et al., 2006). However, the BLa response to BFRE under different levels of applied restriction pressure remains untested. Similarly, the metabolic effect of BFRE is assumed to be associated with the level of hypoxia within the downstream musculature and other tissues (Moritani et al., 1992; Gentil et al., 2006). While hypoxia may be associated with mechanisms of muscle growth through effects on muscle fiber recruitment (Pearson and Hussain, 2015) or tissue growth factors [e.g., hypoxia-inducible factor-1α (HIF-1α)] (Taylor et al., 2016), the measurement of factors associated with hypoxia (e.g., muscle tissue oxygen saturation) remain unclear. Despite one investigation of tissue oxygen saturation across different levels of applied restriction pressure (Downs et al., 2014), this was examined with exercise to failure. As such, it seems pertinent to further examine the metabolic and hypoxic response to BFRE at different levels of applied restriction pressure.

Therefore, the aims of this study were to examine the acute BFRE responses of muscle fatigue, muscle activation, metabolism (BLa, hypoxia via tissue oxygenation) and whole-body cardiopulmonary responses to BFRE with different levels of applied restriction pressure, while also comparing against LLRE and HLRE. We anticipate that an understanding of these acute responses will assist to inform cuff pressure selection for future research that examines chronic BFR training regimens designed to develop muscle strength and size.

## Materials and Methods

### Participants

Ten males (mean ± SD; 25 ± 6 years; 176.8 ± 5.6 cm; 78.14 ± 8.55 kg) were recruited to participate in this study. All were non-smokers who had not participated in any resistance exercise in the past 6 months and were currently undertaking no more than 150 min of physical activity per week. Relatively sedentary participants were selected to minimize variability in baseline MVC torque. In addition, we targeted sedentary participants given our group’s view that BFRE is more relevant and applicable to sedentary cohorts, whether healthy or clinically affected. Of note, we also did not recruit women to reduce the possible variability in cardiovascular function in response to the menstrual cycle. Future research should examine both acute and chronic responses in different sexes. Prior to commencement in the study, participants completed a pre-screening questionnaire and provided written informed consent. Exclusion occurred for any persons with cardiovascular or musculoskeletal conditions or anyone taking medications for cardiovascular or blood pressure control. Participants attended the laboratory at the same time of day for each trial to avoid diurnal influences and were asked to refrain from exercise, and caffeine or alcohol consumption in the 24 h before trials. All subjects provided written informed consent in accordance with the Declaration of Helsinki. The Deakin University Human Ethics and Advisory Group approved this study (HEAG-H 08_2017).

### Study Design

Each participant attended the laboratory on six separate occasions. After an initial familiarization session, five experimental trials were conducted in a balanced, randomized crossover design, in which trials were performed in a random order on separate days, with at least 3 days between trials. The five experimental trials were: a heavy-load trial (80% MVC) without a restriction to blood flow (HL); a low-load trial (20% MVC) without a restriction to blood flow (LL) and; three low-load trials (20% MVC) performed at 40% (B-40), 60% (B-60), and 80% (B-80) of pre-exercise resting limb occlusion pressure (LOP).

### Familiarization

Familiarization served the purpose of determining baseline characteristics for several measurements [muscle thickness (MTH), fat thickness (FTH), maximal whole body oxygen consumption ($\dot{\text{V}}\,{\text{O}}_{2}\,\text{max}$), and MVC] and included standard anthropometric measures [height, body mass, blood pressure (BP), and limb circumference]. MTH and FTH were measured over the *Vastus Lateralis* (*VL*) via 2D ultrasound. A linear array transducer (L12-5) interfaced with an ultrasound system (iU22; Philips Medical Systems, Sydney, NSW, Australia) was placed two-thirds of the way down the line from the anterior superior iliac spine to the lateral side of the patella. MTH and FTH were then measured using on-line calipers in triplicate. Participants then performed a standard incremental cycling test to exhaustion on a cycle ergometer (Lode Excalibur Sport, Lode, Groningen, Netherlands). Expired gases were collected throughout to determine $\dot{\text{V}}\,{\text{O}}_{2}\,\text{max}$. Participants respired through a mouthpiece with nose clip attached, into a standard metabolic cart (Innocor, Innovision A/S, Odense, Denmark). The protocol started at 50 W and then increased by 50 W every 3 min until 9 min, after which the workload increased by 25 W each minute until exhaustion. $\dot{\text{V}}\,{\text{O}}_{2}\,\text{max}$ was defined as the highest whole body oxygen consumption ($\dot{\text{V}}\,{\text{O}}_{2}$) recorded over over a 15-s period during the test. Following a 10-min rest period, participants were seated on an isokinetic dynamometer (Biodex system 4 pro, Biodex Medical Systems, Shirly, United States). Participants were then instructed to perform three unilateral isometric knee extensions at maximal intensity using the dominant leg, with a 1-min rest between each contraction in order to familiarize them with the measurement of maximal voluntary contractile force. Finally, participants completed a short practice of the B-40 trial, performing the first set only to familiarize participants with BFRE and performing each rhythmic isometric contraction at the fixed cadence maintained during the experimental trials (3-s contraction and 1-s relaxation).

### Experimental Trials

**FIGURE 1:**
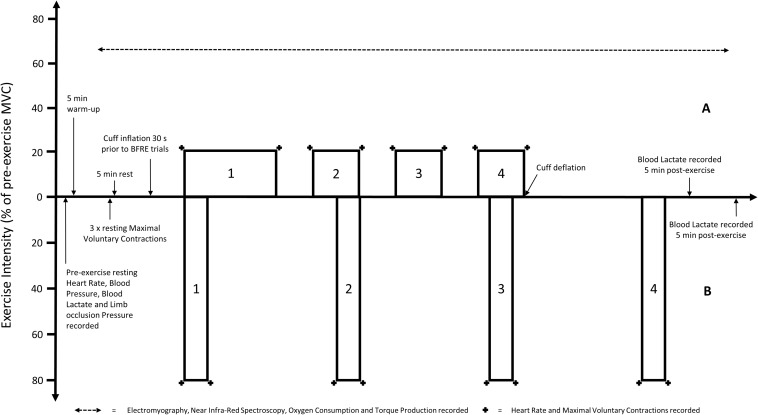
Visual representation of the timing and intensity [based on pre-exercise maximal voluntary contraction (MVC)] of the **(A)** light load resistance exercise trials [including all blood flow restriction exercise (BFRE) trials] and **(B)** heavy load resistance exercise trial. Note that the pre- and post-exercise measurements are not to scale.

All five experimental trials were conducted in a random order, on separate days. Prior to commencement of each trial, participants completed a 5-min warm-up on a cycle ergometer at 75 W. Participants were then seated in an upright position on an isokinetic dynamometer. After all measurement devices were prepared, a total of 3 unilateral isometric knee extensions at maximal intensity were performed using the dominant leg. The greatest MVC was used to determine the load used during the subsequent exercise. The trial began 5 min after the completion of the MVC’s. In all trials, participants performed supervised unilateral rhythmic isometric knee extensions. Repetitions were performed at a fixed cadence (3-s contraction, 1-s relaxation) guided by visual prompts on the Biodex screen, informing participants when to contract and relax, as well as the intensity required to be maintained for each repetition. In each trial, the first and final repetition of each set required participants to perform a MVC. For the LL trial and all BFRE trials, an initial set of 30 repetitions, followed by 3 sets of 15 repetitions were performed with a 30-s rest between each set (Figure 1). For the HL trial, 4 sets of 8 repetitions were performed with 2.5 min rest between each set (Figure 1). Isometric exercise was chosen given the greater suitability for measurement of tissue oxygenation and fatigue during the experimental trials. In combination with the selected duty cycle and target intensity (20%) it was expected that this would not overwhelm any distinguishable effect of the different applied restriction pressures on the variables used to characterize the acute response to BFRE.

### Blood Flow Restriction Trials

In all BFRE trials, participants were fitted with an inflatable pneumatic cuff (86 cm long, 10.5 cm wide: bladder width 8 cm) applied to the most proximal portion of the dominant thigh. The cuff was connected to an automated tourniquet system (A.T.S. 3000, Zimmer, OH, United States) which was used to determine LOP at rest, as previously described (Staunton et al., 2015). LOP was then used to set and adjust the pressure during each BFRE trial. The cuff was inflated to the desired pressure over a 20-s period (40, 60, or 80% LOP) and 10 s later the trial commenced. Pressure was applied continuously throughout each of the sets and during rest periods until the completion of exercise, at which point the cuff was immediately deflated (8 min inflation per BFRE trial).

### Measurements

#### Torque

Torque data was collected throughout the protocol and transmitted as voltage to be recorded in Powerlab 8 (Powerlab 16/35, ADInstruments, Australia). A monitor connected to the Biodex provided participants with visual feedback of the required level of torque when performing contractions. Additionally, verbal feedback/encouragement was given to ensure each participant consistently targeted the required submaximal load and reached maximum values during MVC’s. Following the trial, the MVC torque data was normalized as a percentage of the highest MVC (raw torque) recorded pre-exercise for analysis.

#### Muscle Activity

Muscle activity in the *VL* was measured using EMG throughout the entire protocol. Electrode placement for the *VL* muscle occurred 2/3 of the way down the line from the anterior superior iliac spine to the lateral side of the patella (Hermans et al., 2000). A single ground electrode was fitted on the medial side of the tibial tuberosity. These sites were shaved, then scrubbed with an abrasive gel (Nuprep, Weaver and Company, United States) to remove hair and dead skin cells. The areas were then cleaned with a 70% isopropyl alcohol swab to ensure a clear signal was obtained. Bipolar (2-cm center-to-center) surface EMG electrodes (Ag-AgCl, Foam Electrodes, Covidien, Canada) were used to obtain EMG signals. An analog bandpass filter was applied at a sampling rate of 4000 Hz with a bandwidth of 13 – 1000 Hz. Powerlab was used through an analog-digital interface for analysis. A 500-ms root-mean-square (RMS) value was then analyzed from the plateau of each contraction to indicate muscle activity. The data was then normalized as a percentage of the EMG of the highest MVC (raw torque) recorded pre-exercise for analysis. Prior to analysis, the data were arranged to display both MVC EMG and the average EMG of the final 3 submaximal contractions of each set.

#### Tissue Oxygenation

The level of tissue oxygenation within the *VL* was measured throughout the entirety of each trial by near-infrared spectroscopy (NIRS) using a tissue oximeter sensor (NIRS: Portalite mini 40 mm × 20 mm × 5 mm, Artinis Medical Systems, Netherlands). The NIRS device was placed 2/3 of the way down the line from the greater trochanter to the lateral side of the patella and taped to the leg to ensure that the transmitter-receiver optode distance was always consistent during muscle contraction and/or movement. A black towel was placed over the NIRS device to screen any ambient light. Data was sampled throughout the protocol at a rate of 10 Hz and was recorded as concentration change from baseline in micromoles. A 500-ms average of the samples during each contraction and the immediately following 1 s rest period was recorded. The average of the final 10 s of each set and the average of the middle 15 s of each rest period were recorded for statistical analysis. The variable of interest used for analysis was the “tissue saturation index” (TSI), which is the ratio of signals representing oxy- and deoxy-hemoglobin.

#### Whole-Body Oxygen Consumption

Before each test the O_2_ and carbon dioxide (CO_2_) analysis systems were calibrated using ambient air and a gas of known O_2_ and CO_2_ concentration according to the manufacturer’s instructions. The turbine flow-meter was calibrated using a 3-L syringe. Throughout the entirety of each trial, participants performed open-circuit spirometry, breathing into a mouthpiece with nose clip into a standard metabolic cart. Expired gas measures of $\dot{\text{V}}\,{\text{O}}_{2}$ were averaged into 15-s intervals for analysis.

#### Blood Lactate

Blood lactate was measured from a seated position at rest prior to the commencement of any exercise and then 5 min after the completion of the protocol (to allow for normal circulation of BLa post-deflation in the BFRE trials). BLa was taken via finger-prick on each participant’s non-dominant hand and recorded on a lactate analyzer (Lactate Pro, Arkray Inc., Japan).

#### Heart Rate

Heart rate (HR) was first recorded after 5 min of seated rest. Subsequent HR recordings were taken immediately prior-to and following the first and last contraction of each set, respectively. HR was measured using a standard HR monitor watch receiver with chest strap transmitter (Polar, A300, Polar, NSW, Australia).

#### Blood Pressure

Brachial artery blood pressures [systolic (sBP) and diastolic (dBP)] were measured after 5 min of seated rest before the commencement of each trial. Measurements were taken in the upper arm via automatic auscultation using the non-invasive blood pressure feature on the Innocor system (Innocor, Innovision A/S, Odense, Denmark). Mean arterial pressure (MAP) was also calculated, using sBP, dBP and HR as inputs to determine this value [MAP = dBP + (0.01^*^EXP(4.14 – 40.74/HR)^*^(sBP-dBP))] (Moran et al., 1995).

### Statistical Analysis

All data are presented as mean ± SEM, unless otherwise specified. MVC torque, MVC EMG, submaximal EMG, $\dot{\text{V}}\,{\text{O}}_{2}$ and TSI data were analyzed by a linear mixed model with repeated measures for fixed factors of Trial (HL, LL, B-40, B-60, and B-80) and Time (MVC torque and MVC EMG = Baseline, prior to, and following each set; submaximal EMG = set 1, 2, 3, and 4; $\dot{\text{V}}\,{\text{O}}_{2}$ and TSI = prior to and following each set) followed by pairwise comparisons. BLa and HR data were analyzed using repeated measures analyses of variance (ANOVA) for Trial and Time (BLa = pre- and post-exercise; HR = prior to, and following each set). Additionally, one-way ANOVA’s were run to assess for differences at baseline in all measures between trials. For all analyses, significance was set at *p* < 0.05. All statistical analyses were performed using NCSS [NCSS 12 Statistical Software (2018), NCSS, LLC, Kaysville, Utah, United States].

## Results

### Baseline Characteristics

Participant baseline characteristics and anthropometric data align with the selection criteria, such that participants were relatively physically inactive and demonstrated average fitness ($\dot{\text{V}}\,{\text{O}}_{2}\,\text{max}$) (Table 1). Baseline MVC was not different between trials (Table 2). However, for the HL trial the target torque (80% MVC) achieved by participants was slightly below expected (71 ± 2% MVC) (Table 2), while still being classed as HLRE (American College of Sports Medicine, 2009). Resting LOP was not different between the BFRE trials (Table 2). Moreover, the applied restriction pressure during exercise was progressively greater from B-40 to B-60 to B-80 (*p* < 0.05).

**TABLE 1:** Participant characteristics.

Age (years)	25 ± 6
Height (cm)	176.8 ± 5.6
Body mass (kg)	78.1 ± 8.6
BMI (kg.m^–2^)	24.8 ± 1.6
Systolic blood pressure (mmHg)	114 ± 8
Diastolic blood pressure (mmHg)	66 ± 7
Mean arterial pressure (mmHg)	82 ± 6
Limb circumference (cm)	56.3 ± 3.5
Muscle thickness (cm)	3.47 ± 0.56
Thigh subcutaneous fat thickness (cm)	0.70 ± 0.18
$\dot{\text{V}}\,{\text{O}}_{2}\,\text{max}$ (l.min^–1^)	3.25 ± 0.44
$\dot{\text{V}}\,{\text{O}}_{2}\,\text{max}$ (ml.kg.min^–1^)	41.1 ± 6.8

*Data is presented as Mean ± SD.*

**TABLE 2:** Baseline torque, target torque and restriction pressure for each trial.

**Trial**	**Baseline MVC torque (N.m)**	**Target torque (N.m)**	**Achieved torque (% MVC)**	**LOP (mmHg)**	**Restriction pressure (mmHg)**
LL	155.2 ± 13.5	31.0 ± 2.7	22 ± 1	–	–
HL	158.6 ± 10.4	126.9 ± 8.3^*^	71 ± 2^*^	–	–
B-40	150.6 ± 7.5	30.1 ± 1.5	21 ± 1	227 ± 5	91 ± 2^*^
B-60	139.4 ± 7.2	27.9 ± 1.4	22 ± 0	226 ± 9	136 ± 5^*^
B-80	155.0 ± 9.0	31.0 ± 1.8	20 ± 1	223 ± 7	178 ± 6^*^

Data is presented as mean ± SEM. ^*^Indicates a significant difference from all other trials (*p* < 0.05).

### MVC Torque

**FIGURE 2:**
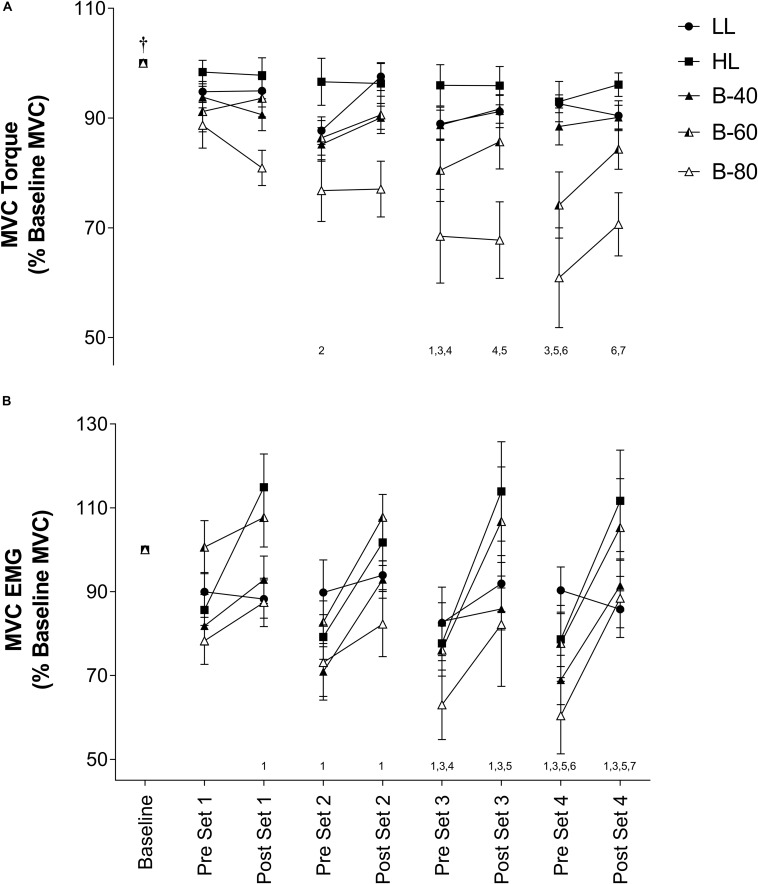
**(A)** Maximal voluntary contraction (MVC) torque and **(B)** MVC electromyography (EMG) in the *Vastus Lateralis* muscle during all five trials [heavy load (HL), light load (LL), 40% limb occlusion pressure (B-40), 60% limb occlusion pressure (B-60), 80% limb occlusion pressure (B-80)]. Values are presented as mean ± SEM. Number values along *x*-axis represent a significant Time effect from the number of time points indicated prior. ^†^Indicates a significant Time effect from all other time points. Significance set at *p* < 0.05.

Torque recorded for each pre- and post-set MVC is shown in Figure 2A. There was a significant main effect for Time such that torque declined progressively across time from baseline. However, without a significant interaction (Time × Trial; *p* = 0.08) this decline was similar between trials, despite a significant main effect for Trial whereby B-80 was significantly lower than all other trials (*p* < 0.001), B-60 was significantly lower than LL and HL but not B-40 (*p* = 0.052), and B-40 was significantly lower than HL, but not LL. There was no difference between HL and LL (*p* = 0.056).

### Muscle Activity

#### Maximal

Electromyography activity for each pre- and post-set MVC is shown in Figure 2B. There was a main effect for Time such that EMG activity significantly increased from pre- to post- for each set, but EMG activity also significantly declined from post-set to the subsequent pre-set. There was no significant interaction (Time × Trial). However, there were several main effects for Trial such that LL (90 ± 5% Baseline MVC EMG) was similar to all trials except B-80 (79 ± 8%), B-80 was also significantly lower than B-60 (96 ± 7%) and HL (96 ± 6%), HL was significantly greater that B-40 (85 ± 5%) and B-40 was significantly lower than B-60 (96 ± 7%).

#### Submaximal

**FIGURE 3:**
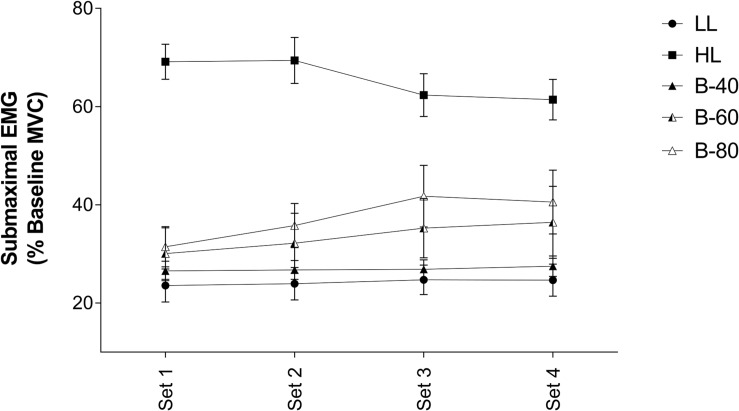
Electromyography (EMG) of submaximal contractions in the *Vastus Lateralis* muscle during all five trials [heavy load (HL), light load (LL), 40% limb occlusion pressure (B-40), 60% limb occlusion pressure (B-60), 80% limb occlusion pressure (B-80)]. Values are presented as mean ± SEM. Significance set at *p* < 0.05.

Average EMG activity over the last 3 submaximal contractions of each set is displayed in Figure 3. Submaximal EMG activity remained constant over time (i.e., no main effect). However, there was a main effect for Trial such that submaximal EMG activity for HL was significantly higher than all other trials (*p* < 0.001), while both B-80 and B-60 were significantly greater than LL (*p* < 0.01). B-80 was also significantly greater than B-40 (*p* < 0.01), although B-60 was not (*p* = 0.0508). There was no significant interaction (Time × Trial).

### Whole Body Oxygen Consumption

**FIGURE 4:**
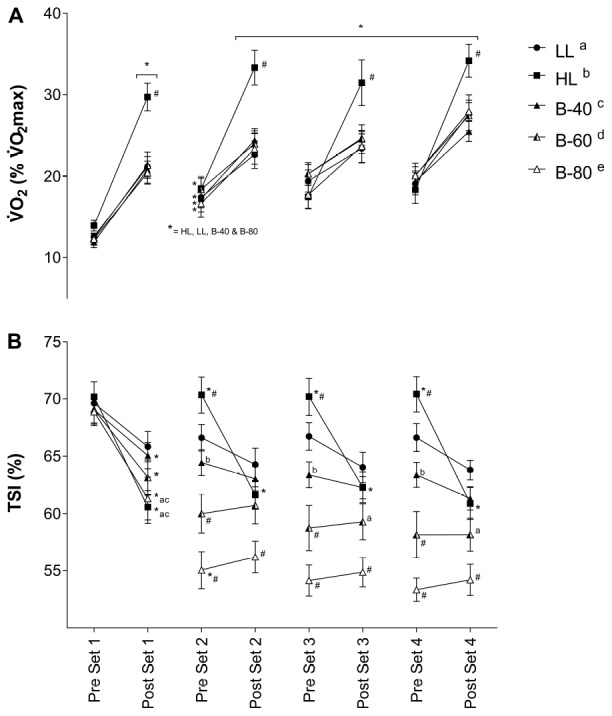
**(A)** Whole body volume of oxygen consumption ($\dot{\text{V}}\,{\text{O}}_{2}$) and **(B)** Muscle oxygenation (TSI) of the *Vastus Lateralis* muscle during all five trials [heavy load (HL), light load (LL), 40% limb occlusion pressure (B-40), 60% limb occlusion pressure (B-60), 80% limb occlusion pressure (B-80)]. Values are presented as mean ± SEM. ^*^Indicates a significant difference from previous time point. Lower case letters (see legend) represent a significant difference between trials at a time point. ^#^Indicates a significant difference from all trials at a time point. Significance set at *p* < 0.05.

Mean whole body $\dot{\text{V}}\,{\text{O}}_{2}$ for the 15-s period pre- and post-set is displayed in Figure 4A. There was a main effect for Time, such that whole body $\dot{\text{V}}\,{\text{O}}_{2}$ increased across all trials (*p* < 0.05). There was also a main effect for Trial, such that submaximal $\dot{\text{V}}\,{\text{O}}_{2}$ was greater in HL than all other trials. A significant interaction (Time × Trial) showed $\dot{\text{V}}\,{\text{O}}_{2}$ for HL to be greater post-set than all other trials (*p* < 0.001), while for all trials, $\dot{\text{V}}\,{\text{O}}_{2}$ increased across all sets and declined during recovery between sets [except from post-set 1 and pre-set 2 for B-60 (*p* = 0.07)].

### Muscle Oxygenation (TSI)

Mean TSI of *VL* for each 15-s period pre- and post-set is displayed in Figure 4B. There was a main effect for Time, and a significant interaction (Time × Trial) such that TSI decreased across the HL, B-40, B-60 and B-80 trials, while TSI remained unchanged across the LL trial. TSI declined significantly across set 1 for the HL, B-40, B-60, and B-80 trials (*p* < 0.05), without a change in the LL trial. While TSI recovered between sets for the HL trial only, TSI also declined across each of the remaining sets in HL only (2, 3, and 4). After declining across set 1 TSI remained unchanged across the three remaining sets for all BFRE trials (Figure 4B). However, in B-80 TSI declined significantly across the recovery period between set 1 and set 2. There was also a main effect for Trial such that TSI for B-80 was significantly lower than all other trials (*p* < 0.001), B-60 was significantly lower than LL, HL and B-40 (*p* < 0.001), and B-40 was significantly lower than HL (*p* < 0.01). In addition, the significant interaction showed TSI for B-80 to be lower than all trials from pre-set 2 onward.

### Blood Lactate

**FIGURE 5:**
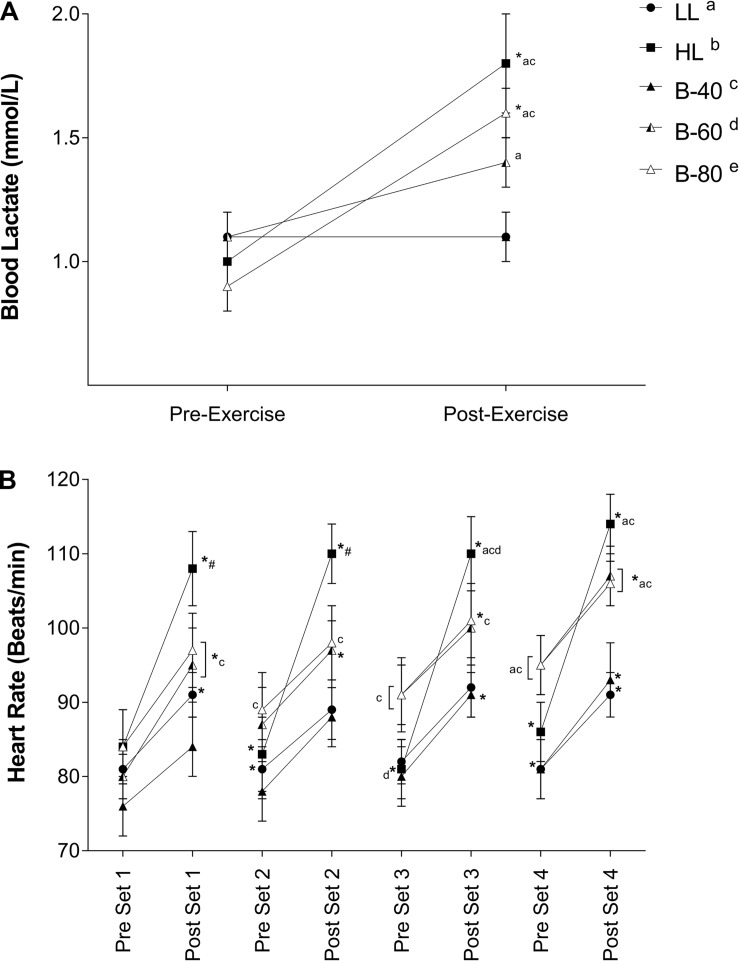
**(A)** Blood lactate concentrations and **(B)** Heart rate during all five trials [heavy load (HL), light load (LL), 40% limb occlusion pressure (B-40), 60% limb occlusion pressure (B-60), 80% limb occlusion pressure (B-80)]. Values are presented as mean ± SEM. ^*^Indicates a significant difference from previous time point. Lower case letters (see legend) represent a significant difference between trials at a time point. ^#^Indicates a significant difference from all trials at a time point. Significance set at *p* < 0.05.

Blood lactate concentration recorded pre- and post-exercise is displayed in Figure 5A. There was a significant main effect for Time and a significant interaction (Time × Trial) such that BLa concentration increased across the HL and B-80 trials, while remaining unchanged across the LL, B-40 and B-60 trials. The significant interaction also showed post-exercise BLa for both HL and B-80 to be greater than both the LL and B-40 trials (*p* < 0.05), while BLa was also significantly greater post-exercise in B-60 compared with LL.

### Heart Rate

Heart rate recorded immediately pre- and post-set is shown in Figure 5B. There was a significant main effect for Time such that HR increased with exercise across each set and declined with recovery between sets. There was also a main effect for Trial such that HR for both HL and B-80 was greater than B-40 (*p* < 0.05). However, a significant interaction showed that post-exercise HR for HL was greater than LL and B-40 in all sets (*p* < 0.05), and greater for B-60 and B-80 than LL and B-40 toward the end of exercise (e.g., pre-set 4 onward).

## Discussion

The present study investigated acute muscle (torque and EMG), metabolic (BLa and TSI) and cardiopulmonary (HR and $\dot{\text{V}}\,{\text{O}}_{2}$) responses to different levels of individualized applied restriction pressure during BFRE. The major finding was that, in general, increases to BFR pressure exaggerated the magnitude of the acute responses to BFRE. As the level of restriction pressure increased the torque and TSI response declined further, while the muscle activity and BLa response was amplified. The outcomes were similar between the B-60 and B-80 trials, and these trials were generally comparable to the HL trial, indicating that BFRE, with a cuff pressure of 60–80% LOP may be a suitable substitute where participants are contraindicated to perform HLRE. However, it is important to recognize that these outcomes are derived from the rhythmic isometric exercise of the present study, and while we expect this to be similar for dynamic exercise, it remains to be tested. In contrast, restriction pressure in the B-40 trial was not sufficient to produce acute responses beyond that of the LL trial.

Despite the target torque (20% MVC) being equivalent among the LL and BFR trials, muscle activity (*submaximal* EMG) required to produce these torques was positively associated with the magnitude of the applied BFR pressure such that muscle activity was lowest in the LL and B-40 trials and increased toward the B-60 then B-80 trials, and finally the HL trial (target torque = 80% MVC) (Figure 3). In contrast, when assessing fatigue across the trials by examining the decline in MVC torque where the B-60 and B-80 trials demonstrated greater fatigue when approaching completion of exercise (Figure 2A), the *maximal* EMG activity was lowest with the greatest restriction pressures (e.g., B-80; Figure 2B). Both these relationships between EMG and torque for *submaximal* and *maximal* responses are supported by previous findings, where increases in the applied restriction pressure led to a progressive increase in submaximal muscle activity and a decrement in maximal torque production (Yasuda et al., 2008; Fatela et al., 2016). The present study is limited by not measuring fatigue via supramaximal electric stimulation, and hence does not inform as to the origin of the observed fatigue, whether central or peripheral (Place et al., 2009). However, the measurement of MVC torque throughout the protocol (i.e., before and after each set) in addition to EMG, provides valuable information around the onset and progression of fatigue developed throughout a BFRE protocol.

Interestingly, there was a decline in MVC torque during the rest periods between some sets toward the end of exercise in both the B-60 and B-80 trials, which also occurred immediately upon cuff inflation prior to the commencement of exercise (Figure 2A). This acute progressive fatigue during the rest periods points substantially to the importance of continued restriction to flow between sets contributing to the potential mechanisms by which BFRE may generate increased muscle strength and size with training in combination with the contractile phase of the activity (Yasuda et al., 2013; Pearson and Hussain, 2015; Iversen et al., 2016). Moritani et al. (1992) previously speculated that deficits in force development may be caused by limited oxygen availability and other blood borne substrates (i.e., glucose and free fatty acids) and/or acidification of the intramuscular environment, with the recruitment of additional motor units serving to compensate for this effect. Therefore, the muscle fatigue, represented by a decrease in MVC torque, and associated increases to muscle activation within the B-60 and B-80 trials may be explained by changes to oxygen availability (TSI) and accumulation of metabolic by-products (lactate).

Muscle tissue oxygenation represented by TSI (Figure 4B) somewhat mirrors fatigue/MVC torque (Figure 2A). However, while fatigue appears progressive across the BFRE bout, in particular for B-80, the decline in TSI is initially rapid across set 1 and maintained from set 2 onward without any sign of recovery. This apparent persistent tissue hypoxia appears more severe with an increase in restriction pressure (B-60 to B-80) while being absent for B-40, so again indicates an effective BFR pressure being between 60 and 80% LOP. Of note, the HL bout also shows a significant and rapid decline in tissue oxygenation, but in this trial there is a rapid recovery of TSI to baseline during rest periods between sets. Visually, this is inverse to $\dot{\text{V}}\,{\text{O}}_{2}$ for HL, and while the BFR and LL trials demonstrate a similar profile but with reduced magnitude for $\dot{\text{V}}\,{\text{O}}_{2}$ (Figure 4A), the lack of recovery of TSI between bouts in the B-60 and B-80 trials suggests that there is a disconnect between whole body $\dot{\text{V}}\,{\text{O}}_{2}$ and the level of tissue oxygenation with BFRE, at least with higher applied pressures that is not observed for HL. It is difficult to suggest whether this may shed light on the mechanism of muscle growth when BFRE is undertaken chronically across a training period. However, it provides evidence to support a hypothesis that BFRE and HL may stimulate muscle adaptation through divergent pathways.

The $\dot{\text{V}}\,{\text{O}}_{2}$ response shows an apparent metabolic demand of the exercise to be entirely dependent on the level of work. That is, HL generates the greatest metabolic demand, with all other trials generating similar but smaller demand independent of the level of BFR applied (i.e., B-40, B-60, and B-80) or not (LL). Although, this is in contrast with others that show BFR to increase metabolic requirements as assessed by $\dot{\text{V}}\,{\text{O}}_{2}$ (Eiken and Bjurstedt, 1987; Abe et al., 2006), where we used individualized pressures and resistance exercise, as opposed to aerobic walking (Abe et al., 2006) or cycling (Eiken and Bjurstedt, 1987) and particularly high pressures (200 mmHg, Abe et al., 2006), or particularly low pressures (50 mmHg, Eiken and Bjurstedt, 1987). However, when examining data for BLa there is a clear relationship between exercising load and the level of glycolytic metabolism that is highest for HL and lowest for LL. We show this to be modulated by the addition of BFR while also being sensitive to the level of the applied restriction such that there is no effect on BLa in the B-40 trial, but a progressive increase in BLa with increasing applied pressure to B-60 and then B-80 (Figure 5A). This aligns with the greater *submaximal* EMG activity with BFR and taken together this lends support for greater Type II fiber recruitment during BFRE (Pearson and Hussain, 2015). To some extent the BLa response also reflects HR (as opposed to $\dot{\text{V}}\,{\text{O}}_{2}$) whereby HL produces the highest HR’s across the trial (again in line with the exercising load) while LL produces the lowest HR’s (Figure 5B). B-40 again shows no evidence of an effect of BFR while B-60 and B-80 increase HR to a similar level to HL by the end of the trial. This response appears to be less graded (i.e., progressively related to the level of applied restriction pressure) but reinforces the suggestion that BFR impacts limb blood flow both during the active exercise periods and importantly during periods of rest between sets. In this respect the present study is limited by not having a direct measure of limb blood flow and, therefore, it is important that this suggestion be confirmed in future research. While we did not measure blood flow *per se* in the present study, our group and others have previously shown the generation of cardiac output for BFRE to be derived from higher HR’s and lower stroke volumes (Brandner et al., 2015; Staunton et al., 2015; May et al., 2017), but the present study adds to this HR data by providing greater resolution to this response by including both rest and exercising HR’s.

### Limitations

One limitation of the present study is that without an objective supramaximal stimulation protocol applied during MVC’s to assess fatigue, we cannot identify the origin of fatigue (central or peripheral) (Place et al., 2009), nor assess the level of volition from participants. However, we did not experience any variation in pre-exercise MVC torque between trials and therefore expect that participant effort was maximal for the pre-exercise measurement. Furthermore, the measurement of both MVC torque and EMG provides some valuable insight into the onset and progression of fatigue throughout a BFRE protocol. BFR may also have an effect on volitional effort during MVC’s throughout exercise and as such it is important to test maximal contractions via supramaximal electrical stimulations in the future to obtain a more objective measure of fatigue. In addition, the present study measured rhythmic isometric contractions which limits extrapolation of the outcomes to more common BFR exercise training regimens. Although as outlined above, given the nature of the responses (i.e., most change occurred during rest), we do not expect the outcomes for more common dynamic BFR exercise to be vastly different.

## Conclusion

The present study characterized the acute muscle (torque and EMG), metabolic (BLa and TSI) and cardiopulmonary (HR and $\dot{\text{V}}\,{\text{O}}_{2}$) responses to different levels of individualized applied restriction pressure during BFRE. While the findings of the present study should not be directly extrapolated to common dynamic modes of BFR exercise, given the graded response to BFR pressures and the nature of the decline in MVC torque, MVC EMG and tissue oxygenation during rest periods, we consider the outcomes of such modes would not be vastly different. We demonstrate these responses to be graded/progressive with increasing applied pressure, from which we speculate that an effective minimum “threshold” of 60% LOP to be necessary for BFRE to be potentially effective with training. In particular we show BFRE at, and above, this 60% LOP threshold to exacerbate fatigue and the metabolic stress of the exercise, while modulating the muscle activity required to complete the work requirements. While these data enable speculation on the possible mechanisms by which BFRE develops skeletal muscle size and strength when undertaken chronically across a training program, such as the importance of the impact of BFR on the rest periods between sets (and possibly between contractions), the outcomes of chronic training regimens using different levels of applied restriction pressures remain to be tested. Overall, notwithstanding the mode of exercise used in the present study, our findings recommend 60–80% LOP as a suitable BFR pressure.

## Ethics Statement

This study was carried out in accordance with the recommendations of the Deakin University Human Ethics Advisory Group. All subjects gave written informed consent in accordance with the Declaration of Helsinki. The protocol was approved by the Deakin University Human Ethics Advisory Group (HEAG-H 08_2017).

## Author Contributions

MI, TR, and SW conceived and designed the study. MI, TR, and MK conducted the data collection. MI, SW, AM, and TR conducted the data analysis. MI, SW, AM, TR, and MK contributed to the writing of the manuscript.

## Conflict of Interest Statement

The authors declare that the research was conducted in the absence of any commercial or financial relationships that could be construed as a potential conflict of interest.
